# Towards universal health coverage: lessons learnt from the COVID-19 pandemic in Africa

**DOI:** 10.11604/pamj.supp.2020.35.2.24769

**Published:** 2020-07-30

**Authors:** Foluke Esther Akinleye, Gbemisola Rebecca Akinbolaji, Joseph Oladimeji Olasupo

**Affiliations:** 1 Department of Clinical Nursing, University College Hospital, Ibadan, Oyo State, Nigeria,; 2 Department of Anatomy, Bowen University, Iwo, Osun State, Nigeria,; 3 Faculty of Pharmacy, University of Ibadan, Ibadan, Oyo State, Nigeria

**Keywords:** Universal health coverage, COVID-19 pandemic, lessons from COVID-19, coronavirus, COVID-19 Africa, African health system, healthcare

## Abstract

With social distancing being a key preventative measure of COVID-19, proper provision of healthcare services becomes a challenge as healthcare professionals are concerned about the risk of potential infection. Telemedicine, a practice that uses telecommunication networks for the delivery of healthcare services and medical education, has been adopted by several countries and has shown to provide positive outcomes. This concept is poorly practiced in African Countries compared to other countries of the world. This paper reiterates the need for the expansion of telemedical systems in Africa for the dual goals of COVID-19 prevention and provision of quality healthcare services to people.

## Commentary

Africa and the rest of the world are experiencing an outbreak of a novel beta-coronavirus known as severe acute respiratory syndrome coronavirus 2 (SARS-CoV-2), first detected in Wuhan city, China. This virus (SARS-CoV-2) caused the coronavirus disease 2019 (COVID-19) and was declared a pandemic by the World Health Organization (WHO) on 11th March, 2020 [1]. COVID-19 has not only affected the health and caused the death of many people; it has also affected virtually all aspects of human life. After an upsurge in the number of confirmed COVID-19 cases in many countries of the world, country-wide and local quarantines have been imposed, restricting activities that will result in a large gathering of people (travel, weddings, worship in churches and mosques, school, work, leisure, and other aspects of life of the citizens). In Africa, Rwanda, Tunisia, Nigeria, and South Africa were the first countries to declare the lockdown as a means of curbing the spread of the COVID-19. Other countries have since joined the train [2]. The pandemic has however affected the way the health institutions operate. For instance, some hospitals have reorganized the hospital networks based on a hub-and-spoke design. Pharmaceutical care, medical care, and nursing care have taken a modified form so as to protect the healthcare worker(s) from getting infected; people with chronic diseases such as cancer, diabetes and hypertension have their routine screening suspended and those with early and advanced cases treated as outpatients [3,4].

As the pandemic expands globally, the burden on Africa´s healthcare systems that has been increasingly overwhelmed needs to be eased. Globally, patients have concerns about seeking medical help for conditions that are non-COVID-19 related and non-urgent. This is due to the need to observe social distancing and concerns of contracting COVID-19 [5]. The healthcare professionals as well, are apprehensive about providing quality conventional care, consequently affecting the level of care patients get for their medical conditions. It seems all the focus on healthcare has gone to COVID-19 while other health needs of the population are being given much less attention. If the problem of inaccessibility to healthcare services is not addressed, access to certain types of care would be reduced. If access to care is reduced, people´s health is likely to suffer. With the difficulty experienced by patients in need of medical care, some resort to the use of herbal remedies, or self-medication, neglecting the potential damages by these acts-poisoning and complication due to the use of drugs not recommended by qualified personnel. Then there is the greatest danger of all; deaths resulting from neglect of individuals by hospitals [6]. It is very important that there is unrestricted access to healthcare in the face of the COVID-19 pandemic but this is not without challenges. There is this crucial need for a feasible approach and intervention that will address the problem of inaccessibility to healthcare in light of the COVID-19 pandemic. In this commentary, we try to bring an important concept that would help achieve the dual goals of COVID-19 containment and provision of healthcare services to patients regardless of their location.

To ensure healthy lives and promote wellbeing amidst this unprecedented event, a telemedical approach needs to be implemented in developing countries. Telemedicine bridges the gap between patients and quality healthcare services, thus enabling anyone with an internet connection or mobile phone receive adequate care regardless of their location. Video consultation and chats are some of the mediums that telemedicine leverages to ensure a good healthcare service delivery [7]. It is worrisome to know that the burden on the health system caused by shortage of healthcare professionals in Africa has been worsened by the emergence of COVID-19 pandemic. Thirty-one (31) out of the 54 African countries have a doctor-patient ratio of about 1:10,000 compared to the favourable ratio in developed countries like Germany (1:417) and Italy (1:270) [8]. Through telemedicine, patients can get access to healthcare professionals around and outside the country, thereby minimizing the effect caused by this obvious shortage of healthcare professionals and social distancing.

After recording few cases of COVID-19, some African countries such as Mali, Botswana, Uganda, Senegal, South Africa, and Ghana advised patients to seek medical attention online rather than physical consultations [9,10]. However, this telemedical medical network which was aimed at filling the gap in the inaccessibility of medical care by patients came with its own challenges. Poor internet connection, lack of education about telemedicine, instability of basic infrastructure with special emphasis on the electric supply in Africa top the ladder of challenges faced by African countries in the establishment of a good telemedical network. The limited knowledge possessed by Africans about telemedicine is one of the reasons why it is still unpopular in many African countries. Physical contact with healthcare professionals is still needed because there´s a limit to how far remote doctors can go in treating illnesses.

## Conclusion

The COVID-19 outbreak has caused unprecedented disruption to humans’ activities including healthcare delivery. This impact can reverberate in African healthcare institutions much longer after the outbreak has been finally controlled. However, with the right measures put in place and approaches such as telemedicine taken, the effect would not adversely affect the quality of life of the people. As African countries and others around the world weigh new policies in a bid to contain the spread of the virus and provide medical care, there is need to consider the creation of a well programmed telemedical system or the expansion of existing ones; a necessary step towards increasing accessibility to healthcare services during the COVID-19 pandemic.
